# Not Only Soldiers Have Weapons: Evolution of the Frontal Gland in Imagoes of the Termite Families Rhinotermitidae and Serritermitidae

**DOI:** 10.1371/journal.pone.0015761

**Published:** 2010-12-30

**Authors:** Jan Šobotník, Thomas Bourguignon, Robert Hanus, David Sillam-Dussès, Jitka Pflegerová, František Weyda, Kateřina Kutalová, Blahoslava Vytisková, Yves Roisin

**Affiliations:** 1 Research Team of Infochemicals, Institute of Organic Chemistry and Biochemistry, Czech Academy of Sciences, Prague, Czech Republic; 2 Evolutionary Biology and Ecology, CP 160/12, Université Libre de Bruxelles, Brussels, Belgium; 3 Department of Physiology, Institute of Entomology, Biology Centre, Czech Academy of Sciences, České Budějovice, Czech Republic; 4 Faculty of Science, Charles University in Prague, Prague, Czech Republic; 5 University of Life Sciences, Faculty of Forestry and Wood Sciences, Department of Forest Protection and Game Management, Prague, Czech Republic; Ghent University, Belgium

## Abstract

**Background:**

The frontal gland is a unique adaptation of advanced termite families. It has been intensively studied in soldiers with respect to its anatomy and chemistry, with numerous novel compounds being discovered within the tremendous richness of identified products. At the same time, the presence of the frontal gland in non-soldier castes received only negligible attention in the past.

**Principal Findings:**

Here, we report on the development of the frontal gland in alate imagoes of 10 genera and 13 species of Rhinotermitidae and Serritermitidae, in order to shed light on the evolution and function of this gland in imagoes. All investigated species possess a frontal gland. In most cases, it is well-developed and equipped with a sac-like reservoir, located in the postero-dorsal part of cranium, but reaching as far as the seventh abdominal segment in some Rhinotermitinae. The only exception is the genus *Psammotermes*, in which the gland is very small and devoid of the reservoir.

**Conclusions:**

Our direct observations and comparisons with soldiers suggest a defensive role of the gland in imagoes of all studied species. This functional analogy, along with the anatomic homology between the frontal gland in soldiers and imagoes, make it likely that the gland appeared once during the early evolution of rhinotermitid ancestors, and remained as a defensive organ of prime importance in both, soldiers and imagoes.

## Introduction

Termites are an abundant group of decomposers; they are dominant arthropods in tropical regions and therefore also subject to intense competition and predation by various animal taxa [1], [2]. They developed many strategies to protect themselves against opponents, including a specialised defensive caste of soldiers, ancestral to all termites [3]. Soldiers, originally with enlarged biting mandibles [4], subsequently evolved into a multitude of shapes and functions, including the snapping soldiers in the *Termes-Capritermes* group, able to produce one of the fastest ever recorded biological accelerations with their mandibles [5]. These mechanical ways of defence are often coupled with chemical weaponries involving frontal, labial and labral glands [1], [6]–[8]. Mechanical defence strategies were reduced or even completely lost in some groups, such as in small soldiers of Rhinotermitinae or in all soldiers of Nasutitermitinae, whose mandibles are strongly reduced and whose defence entirely rests upon the secretion of the enlarged frontal gland with specific delivery mechanisms [1].

The frontal gland in soldiers is a large, unpaired organ without any equivalent in other insects [9]. Numerous studies were devoted to the chemistry of the frontal gland in soldiers, discovering the tremendous chemical diversity of produced compounds, with many of them being novel. From a functional point of view, these compounds can act in concert in a multitude of defensive roles, such as contact poisons, glues, anti-healants, repellents or alarm pheromones [for review see 1,6,8]. While the anatomy of the frontal gland has been studied in soldiers of numerous species, its presence in other castes received less attention, although it is known in presoldiers [10]–[13], imagoes [9], [13]–[16], and workers [9], [17]. The frontal gland is developed as a sac-like organ in soldiers and presoldiers, either filling a large part of the head, such as in many Termitidae [9], or extending deep into the abdomen, such as in Rhinotermitidae and Serritermitidae [9], [13], [18]. In soldiers, the secretory epithelium is usually composed of class 1 cells (according to the classification of Noirot & Quennedey [19]), except for *Coptotermes*, in which both class 1 and 3 secretory cells occur [7]. By contrast, it is never a sac-like organ in workers, in which it only occurs as an epidermal thickening [9], [17].

In imagoes, the frontal gland is present either as a sac-like organ (*Prorhinotermes* [13], *Reticulitermes* [15], *Heterotermes* and *Rhinotermes* [14], *Odontotermes* [16], *Macrotermes* [9]) or as an epidermal thickening (*Nasutitermes* [14], *Cubitermes* and *Termes* [9]). Unfortunately, only superficial information (schematic drawing or only a brief description) is available for all above-mentioned species except *Prorhinotermes simplex*, in which the frontal gland ultrastructure was compared among castes [13]. The imaginal frontal gland secretion of *Prorhinotermes* spp. consists of toxic and irritant compounds and thus likely fulfils a defensive function as in soldiers [20]. The formation of the frontal gland takes place during the imaginal moult of the last nymphal instar in *Reticulitermes* [9], while it is already formed at the end of the single nymphal instar of *Prorhinotermes* [21].

The frontal gland represents an important synapomorphy of a clade comprising Serritermitidae + Rhinotermitidae + Termitidae [9], [14]. Whereas the Termitidae family forms a monophylum nested within the Rhinotermitidae, the position of the Serritermitidae is still unresolved: they could either be the sister group of the Rhinotermitidae or be nested within them [22]–[24]. The precise phylogenetic arrangement of genera is far from being fully resolved, but molecular studies consistently support Rhinotermitinae as a monophyletic group which originated early in the evolution of the family [22], [23]. The monophyly of Heterotermitinae + Coptotermitinae is also supported and this group is placed as the sister group of Termitidae [22], [23], though this scenario deserves further investigation [24]. Finally, three other genera, namely *Prorhinotermes*, *Termitogeton* and *Psammotermes*, are basal lineages with uncertain relationships [22]–[25].

In soldiers of many termite species in the families Rhinotermitidae, Serritermitidae and Termitidae, the frontal gland is a prominent organ, which often constitutes their major or only weapon. Being a spectacular defensive device in a spectacular defensive caste, the frontal apparatus of soldiers has received much attention, but this organ is still poorly known in other castes. In the present work, we study the anatomical evolution of the frontal gland in imagoes of 10 genera and 13 species of Rhinotermitidae and Serritermitidae, which allows us to envision possible scenarios of frontal gland evolution in termites.

## Results

**Figure 1:**
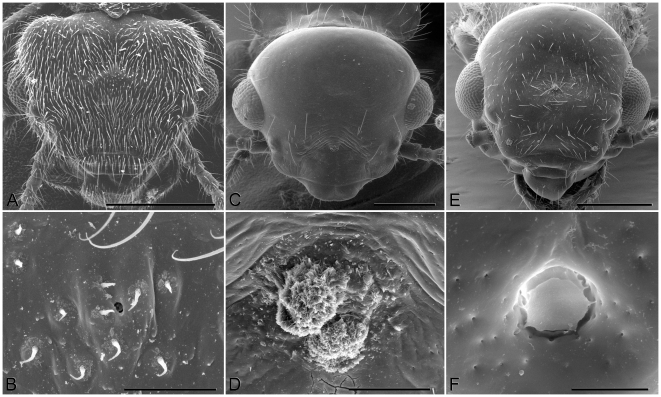
Scanning electron microscopy pictures of heads of alate imagoes. *Termitogeton planus* alate head (A) and its fontanelle (B); *Schedorhinotermes dimorphus* Desneux, alate head (C) and its fontanelle (D); *Coptotermes remotus* Hill, alate head (E) and its fontanelle (F). Scale bars: 1A = 500 µm, 1B = 20 µm, 1C = 500 µm, 1D  = 100 µm, 1E  = 500 µm, 1F  = 10 µm.

The opening of the frontal gland (fontanelle) of imagoes is a simple rounded pore located at the top of the head, very narrow (2 µm) in *Termitogeton* (Fig. 1), but more than 10 µm wide in other genera (Fig. 1). Its location is variable, from a very anterior position in Rhinotermitinae, to a more posterior position in most other genera (see Table 1). The frontal gland is always supplemented by a set of class 3 secretory cells (*sensu* Noirot & Quennedey [19]) which release their products in the vicinity of the fontanelle (then their openings are visible on the frons in SEM), or directly into the fontanelle (then they may be seen in sections, but not in SEM, like e.g. in *Termitogeton*).

**Table 1:** Dimensions and relative size of the frontal gland (FG) and fontanelle position in the studied specimens.

Species	Sex	Total FG volume (mm^3^)	Head length (mm)	Relative FG volume	Epithelium thickness (µm)	Relative fontanelle position
*Glossotermes oculatus*	♂	0.0015	0.76	5.15	29–115	0.5
	♀	0.0012–0.0022	0.7	5.28–9.67	21–93	0.45
*Termitogeton planus*	♂	0.001–0.0013	0.54–0.56	9.58–11.2	27–84	0.58
	♀	0.00066	0.59	4.85	25–39	0.57
*Psammotermes hybostoma*	♂	0.0005–0.0008	0.86–0.93	1.19–1.5	29–78	0.51
	♀	0.0005–0.0008	0.91–0.96	1–1.37	44–90	0.5
*Psammotermes allocerus*	♂	0.0018	0.87	4.12	69–122	0.54
	♀	0.001	0.84	2.55	69–77	0.55
*Parrhinotermes browni*	♂	0.1069	0.99	166	10–29	0.29
*Schedorhinotermes translucens*	♂	0.5685	1.4	312	16–32	0.29
	♀	0.1544	1.51	67.6	15–25	0.28
*Dolichorhinotermes longilabius*	♂	0.3028	0.91	606	7–22	0.24
	♀	0.5308–0.5686	0.98–1.01	832–850	21–56	0.25
*Rhinotermes* sp.	♂	0.434–0.7033	1.39–1.44	244–355	14–44	0.24
	♀	0.7485	1.37–1.49	341–439	19–40	0.22
*Reticulitermes lucifugus*	♂	0.006	0.99	9.32	40–197	0.51
	♀	0.0069	1	10.4	51–142	0.5
*Heterotermes tenuis*	♂	0.0023–0.0044	0.94–0.96	4.18–7.5	7–26	0.44
	♀	0.0243	1.01	35.6	18–38	0.45
*Heterotermes paradoxus*	♂	0.043–0.0443	0.96–1.01	64.8–73.3	5–17	0.5
	♀	0.0884–0.0944	1.01–1.02	129–134	15–47	0.47
*Coptotermes formosanus*	♂	0.011	1.17	10.4	8–15	0.45
	♀	0.01	1.22	8.3	13–16	0.45
*Coptotermes testaceus*	♂	0.0026–0.0038	1.18–1.24	2.39–3.01	8–29	0.46
	♀	0.003–0.0046	1.1	3.4–5.21	14–33	0.47

**Figure 2:**
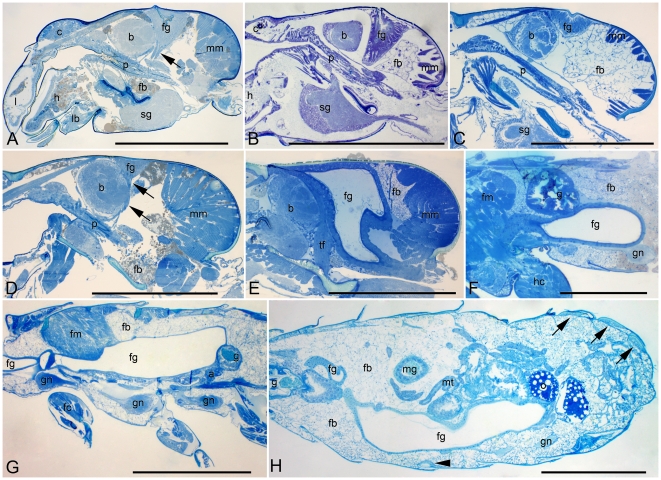
The development of the frontal gland in Serritermitidae and Rhinotermitidae: Termitogetoninae, Psammotermitinae, and Rhinotermitinae. A: *Glossotermes oculatus*, female head. Arrow marks tentorial-fontanellar muscle. Bar represents 0.5 mm. B: *Termitogeton planus*, male head. Bar represents 0.5 mm. C: *Psammotermes allocerus*, female head. Bar represents 0.5 mm. D: *Psammotermes hybostoma*, male head. Arrows mark tentorial-fontanellar muscle. Bar represents 0.5 mm. E: *Dolichorhinotermes longilabius*, female head. Bar represents 0.5 mm. F: *Dolichorhinotermes longilabius*, female thorax-abdomen. Bar represents 0.5 mm. G: *Parrhinotermes browni*, male thorax. Bar represents 1 mm. H: *Rhinotermes* sp., female abdomen. Arrowhead marks sternal gland, arrows mark tergal glands. Bar represents 1 mm. Abbreviations: a, acini of labial gland; b, brain (supraoesophageal ganglion); c, clypeus; fb, fat body; fc, fore coxa; fg, frontal gland; fm, flight muscles; g, gizzard; gn, ganglion of neural cord; h, hypopharynx; hc, hind coxa; l, labrum; lb, labium; mg, midgut; mm, mandibular muscles; mt, malpighian tubules; o, oocyte; p, pharynx; sg, suboesophageal ganglion; tf, tentorial-fontanellar muscle.

**Figure 3:**
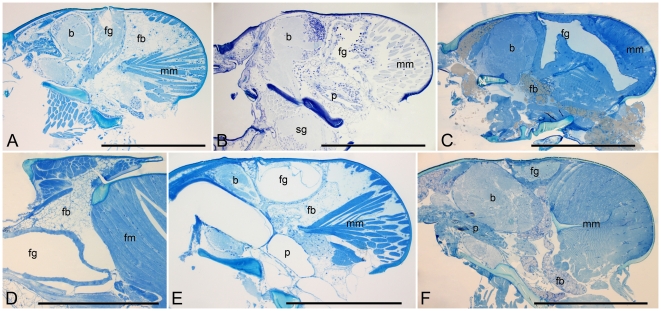
The development of the frontal gland in Rhinotermitidae: Heterotermitinae, and Coptotermitinae. A: *Reticulitermes lucifugus*, male head. B: *Heterotermes paradoxus*, male head. C: *Heterotermes tenuis*, female head. D: *Heterotermes paradoxus*, female thorax. E: *Coptotermes formosanus*, male head. F: *Coptotermes testaceus*, male head. Bar represents 0.5 mm in all figures. Abbreviations: b, brain (supraoesophageal ganglion); fb, fat body; fg, frontal gland; fm, flight muscles; mm, mandibular muscles; p, pharynx; sg, suboesophageal ganglion.

The frontal gland of *Glossotermes oculatus* (Fig. 2A) is developed similarly in both sexes. It is small, squeezed dorsally between the brain and mandibular muscles, with a tiny reservoir opening above the posterior part of the brain. A similar development occurs in *Termitogeton planus* (Fig. 2B), in which a small frontal gland is made of columnar cells and a fairly small reservoir opening behind the brain. *Reticulitermes lucifugus* also reveals a similar development of the frontal gland (Fig. 3A), which is slightly larger, reaching ventrally the pharynx.

In *Psammotermes allocerus* (Fig. 2C), the frontal gland epithelium is also composed of columnar cells but the reservoir is reduced to a shallow depression in the head surface. This depression is missing in the related *P. hybostoma* (Fig. 2D), in which no trace of a reservoir can be found.

In all studied Rhinotermitinae, the frontal gland development is nearly identical. It is a huge sac filling a considerable part of the cranium (Fig. 2E), and continuing as a thick tubular structure usually into the abdomen (Fig. 2F, 2G, 2H). The extent of the reservoir in the abdomen of Rhinotermitinae imagoes is variable, even between individuals of the same species and sex. The reservoir usually ends in the third or fourth abdominal segment, but the gland sometimes does not reach the abdomen (in *Parrhinotermes* male, see Fig. 2G) or reaches only the first abdominal segment (one of two *Dolichorhinotermes* females; see Fig. 2F, *Schedorhinotermes* female), while it reaches as far as the 7th abdominal segment in *Rhinotermes* females (Fig. 2H). The reservoir diameter decreases in the cervix and slowly increases up to the end. The large reservoir is surrounded by cuboidal secretory cells and opens through the fontanelle above the anterior part of the brain, more anteriorly compared to other groups (see Table 1).

In *Heterotermes tenuis*, the frontal gland is a large sac occupying nearly the whole posterior part of the cranium and pushing the mandibular muscles backwards (Fig. 3C). The fontanelle is situated just above the posterior brain. The secretory epithelium is thinner in males (compare Fig. 3B and Fig. 3C), and the reservoir is many times folded, while it is more voluminous in females. A similar situation was observed in *Heterotermes paradoxus*, where the gland is even larger (in both sexes), reaching the anterior mesothorax (ending at the anterior part of the indirect dorso-ventral flying muscles; Fig. 3D).

In *Coptotermes testaceus*, the frontal gland is rather small, surrounded by a thin layer of secretory cells (Fig. 3F). The gland is compressed between the brain and mandibular muscles, and the fontanelle is placed above the middle of the brain. The same is true for *Coptotermes formosanus*, in which the frontal gland is slightly larger, mainly due to the larger amount of secretion stored (Fig. 3E).

## Discussion

Overall, we studied the frontal gland in alate imagoes of 13 species from 10 genera representing the major lineages of Rhinotermitidae and Serritermitidae. All of them possess a well developed and functional frontal gland, equipped with a reservoir in all species but *Psammotermes* spp. The gland is very large in all Rhinotermitinae as well as in *Heterotermes paradoxus*, where it extends out from the head to the thorax or abdomen. The similar development of the frontal gland in all Rhinotermitinae studied (particular species differ only by the posterior extent of the reservoir) provides further evidence of the monophyletic origin of this taxon. Although filling a large part of the posterior head, the frontal gland is smaller in *Heterotermes tenuis* and *Prorhinotermes simplex* [13], while it is reduced in size in *Glossotermes*, *Termitogeton*, *Reticulitermes* and *Coptotermes*. Finally, the gland is greatly reduced in *Psammotermes*, where the reservoir disappears making thus the functional frontal gland reservoir-free.

The frontal gland development is not always conserved between sexes and among related species. The gland is developed similarly in both *Heterotermes paradoxus* and *H. tenuis*, but it appears smaller and partially folded, confined to the head in the latter species. In both cases, alates were collected inside their wood-shelter before swarming, so that the gland volume might considerably increase before the alates get ready to fly out, possibly reaching the same extent in *H. tenuis* and *H. paradoxus*. Additionally, sex differences occur in both *Heterotermes* species, with females having an epithelium made of cuboid cells, while rather squamous cells occur in males. However, these sex differences are not linked with the composition of the frontal gland secretion, which is very similar in both sexes of *H. tenuis* (Krasulová, unpublished). The frontal gland is also larger in *Coptotermes formosanus* than in *C. testaceus*, due to reservoir shrinking in the latter species. As imagoes of both species were collected during their dispersal flight, this dissimilarity likely reflects natural differences between the species. These observations suggest that the overall development of the frontal gland can change quite rapidly during evolution, possibly according to ecological factors and the trade-off between the number of alates produced by the colony, and their chemical protection against the local pool of predators and the pressure they exert on them.

The overall size of imaginal frontal gland varies considerably among genera, and is not always correlated with the gland size in soldiers (see Table 2). Indeed, *Glossotermes* and *Coptotermes* have huge frontal glands in soldiers [7], [18], but rather small glands in alates. Also, the opposite example can be found; in *Heterotermes* spp., the gland is rather small in soldiers (Šobotník & Bourguignon, unpublished) while it is fairly large in imagoes. This differential development of the frontal gland in imagoes and soldiers suggests distinct caste-specific evolutionary routes of the frontal gland development after its origin in the common ancestor of rhinotermitids and serritermitids. The most striking observation is the absence of the sac-like structure of the frontal gland in alates of *Psammotermes* and some Termitidae [9], [14], while it always remains sac-like in the soldier caste. All these findings suggest that the frontal gland of termite imagoes is by no means an ontogenetic by-product of the selected expression of the soldier frontal gland in imagoes, but rather an adaptive organ of probably great value, resulting from caste-specific selective pressures.

**Table 2:** Comparison of the frontal gland development between soldiers and imagoes.

		Frontal gland in imagoes		
		Small	Medium	Large
Frontal gland in soldiers	Small	*Psammotermes* spp., *Termitogeton planus*, *Reticulitermes lucifugus*	*Heterotermes tenuis*	*Heterotermes paradoxus*
	Large	*Glossotermes oculatus* [18], *Coptotermes* spp.,	*Prorhinotermes simplex* [13]	*Parrhinotermes browni*, *Dolichorhinotermes longilabius*, *Schedorhinotermes translucens*, *Rhinotermes* sp.

Footnote: Size (small, medium, large) refers to relative size compared to members of the same caste in other species. The data about frontal gland size in soldiers are adapted from [7] or are based on unpublished observations by Šobotník & Bourguignon. The gland size in soldiers refers to minor soldiers in soldier-dimorphic species of Rhinotermitinae.

The frontal gland epithelium of soldiers is formed only by class 1 cells, except for *Coptotermes* in which both class 1 and 3 cells occur [7]. Our TEM observations (Šobotník & Kutalová, unpublished) showed that this latter arrangement also occurs in alates of *Coptotermes* and *Heterotermes*, providing thus an interesting synapomorphy of these two sister genera [22]. Class 3 cells, located in the vicinity of the fontanelle (but not included into the secretory epithelium) share the same ultrastructure than other class 3 cells scattered all over the body. Therefore, they are not likely to participate directly in the function of the frontal gland [8].

An interesting question is the mode of action of the frontal gland in terms of control over the secretion release. In all studied species, there is a pair of tentorial-fontanellar muscles attached to the frontal gland epithelium in the ventro-anterior part of the cephalic epithelium. These muscles even occur in all species and castes of termites studied so far [9,14, Šobotník, unpublished], without any clear function. We hypothesise that the reservoir is emptied by increase of body pressure (by contraction of intersegmental muscles), and the tentorial-fontanellar muscles prevent the plugging of the fontanelle by the secretory epithelium due to the increase of pressure, by stretching the epithelium downwards (see Fig. 2E). The same mechanism is expected to take place in soldiers as well, as the tentorial-fontanellar muscles are arranged similarly in the majority of the studied species [9,13, Šobotník, unpublished].

Termite reproductives are defended by their nestmates all over their life, except for a short period between the dispersal flight and new colony establishment. During this period, they experience strong predation from various animal taxa [see 11,26]. Termite imagoes have therefore developed strategies to increase their chances to survive the dispersal and initial phases of the colony establishment. One of them is the staging of short and synchronized dispersal flights of innumerable imagoes, which saturate predators. Chemical defence has also been proven in *Prorhinotermes*, whose imaginal frontal gland produces several defensive compounds [20], including toxic nitrocompounds [27] and sesquiterpenes, frequently cited as irritants [see e.g. 6,28,29]. The amount of defensive compounds present in the frontal gland reservoir peaks at the time of swarming, and drops to zero when the first soldier offspring appears [20]. There are also observations of unpalatability of termite imagoes without any obvious reason [7]. Indeed, some of us (Bourguignon, Cvačka & Šobotník, unpublished) observed that all *Coptotermes testaceus* alates collected by the wasp *Polybia scrobalis surinama* were decapitated prior to be stored in the nest, while alates of *Anoplotermes s.lat.* spp. (which only possess a tiny frontal gland without reservoir; Šobotník, unpublished) were not. This confirms the functional significance of the frontal gland, which can be removed by small predators, while larger ones do not have this option and might be repelled after eating a few alates. One can thus expect that imagoes equipped with the frontal gland might limit the predation on relatives flying out of the nest. We hypothesise a trade-off between the number of alates released and the investment in their chemical defence; those endowed with a large and costly frontal gland would be efficiently protected, whereas alates without it could be produced in higher numbers but would face a higher risk of predation.

## Materials and Methods

In order to acquire specimens of most rhinotermitid and serritermitid genera, samples were collected across four continents, namely in French Guiana, New Guinea, Egypt, South Africa and Italy, either in fixative or in alcohol (see Table 3).

**Table 3:** List of the specimens studied and their origin.

Species	Conservation method	Locality	Date	Number of specimens studied
*Glossotermes oculatus* Emerson	Fixative	Petit Saut, French Guiana	2.ii.2008	2 ♂, 3 ♀
*Psammotermes hybostoma* Desneux	Fixative	Ezbet Dush, Al Wadi al Jadid, Egypt	24.iii.2010	2 ♂, 2 ♀
*Psammotermes allocerus* Silvestri	Alcohol	Namaqualand, South Africa	24.iv.1918	1 ♂, 1 ♀
*Termitogeton planus* Haviland	Alcohol	50 km S of Nabire, Papua, Indonesia	xi.1995	2 ♂, 1 ♀
*Reticulitermes lucifugus* (Rossi)	Alcohol	Colony collected at 21.iv.1998 near Alberese (10 km S of Grosseto, Italy),	19.v.1998	1 ♂, 1 ♀
*Heterotermes tenuis* (Hagen)	Fixative	Petit Saut, French Guiana	8.i.2010	4 ♂, 1 ♀
*Heterotermes paradoxus* (Froggatt)	Alcohol	Kaimana, Papua, Indonesia	xi.1995	2 ♂, 2 ♀
*Coptotermes formosanus* Shiraki	Alcohol	Lab colony collected in Hsin-hui, Kuang-chou (Canton) province, China in 1963	iii.1997	1 ♂, 1 ♀
*Coptotermes testaceus* (Linnaeus)	Fixative	Petit Saut, French Guiana	2.ii.2008	3 ♂, 2 ♀
*Parrhinotermes browni* (Harris)	Alcohol	Yapsiei, Papua New Guinea	11.iii.1994	1 ♂
*Dolichorhinotermes longilabius* (Emerson)	Fixative	Petit Saut, French Guiana	8.i.2010	1 ♂, 2 ♀
*Schedorhinotermes translucens* (Haviland)	Alcohol	Pimaga, Papua New Guinea	18.x.1988	1 ♂, 1 ♀
*Rhinotermes* (Hagen) sp.	Alcohol	Petit Saut, French Guiana	23.i.2007	2 ♂, 2 ♀

When living termites were available, they were submerged into a drop of fixative (2.5% glutaraldehyde in 0.1 M cacodylate buffer at pH 7.2), and the body was cut into head (mandibles carefully removed), thorax and abdomen. After one day of fixation at 4°C, the samples were washed with 5% glucose in 0.1 M cacodylate buffer which was exchanged every 10 days and stored at 4°C until further steps. The tissues were postfixed for 2 hours in 1.5% osmium tetroxide in a 0.1 M cacodylate buffer and dehydrated with an ethanol series. Tissues were embedded into standard Spurr resin. 1 µm thick sections were cut with an Ultracut Reichert-Jung, stained with either Azure II or Methylene blue solutions, and studied using a Carl Zeiss Amplival optical microscope equipped with a Canon EOS 500D camera.

Some samples were obtained from termite collections, where they were stored in 80% ethanol since collection in the field. The body was cut into parts and placed into 99.9% ethanol for 1 day at 4°C. The samples were embedded into Spurr resin and handled as described above.

For scanning electron microscopy, specimens were dehydrated through immersion in a standard ethanol series, impregnated for 24 hours in hexamethyldisilazane, air dried and gold coated. Microphotographs were taken with a Philips XL 30 ESEM.

The reservoir surface (S) of the frontal gland was measured from sagittal section images with the software ImageJ, while its width (W) was estimated from the number of parasagittal sections on which the frontal gland occurred. The volume (V) was then estimated by the equation: V = 2/3×S×W, based on the formula for the volume of an asymmetric ellipsoid. Whenever the gland reservoir extended in the thorax or abdomen, volumes calculated from head, thorax and abdomen were merged. The size of the frontal gland relative to the body size was expressed as V/L^3^, where L is the head length, calculated as the distance between the clypeo-frontal suture and the posterior margin of the head. The values were then converted into multiples of the smallest observed value. To describe the relative position of the fontanelle, we related the distance between the clypeo-frontal suture and the fontanelle to the head length. These measurements and calculations were performed with all studied specimens (for overview see Table 3).
